# Measuring the Impact of Bilingualism on Executive Functioning Via Inhibitory Control Abilities in Autistic Children

**DOI:** 10.1007/s10803-021-05234-y

**Published:** 2021-08-18

**Authors:** Lewis Montgomery, Vicky Chondrogianni, Sue Fletcher-Watson, Hugh Rabagliati, Antonella Sorace, Rachael Davis

**Affiliations:** 1 The Salvesen Mindroom Research Centre, University of Edinburgh, 5th Floor, Kennedy Tower, Morningside Place, Edinburgh, EH10 5HF UK; 2 School of Philosophy, Psychology, and Language Sciences, University of Edinburgh, Edinburgh, EH8 9AD UK

**Keywords:** Executive functioning, Autism, Bilingualism, Inhibitory control, Second language exposure, Cognition

## Abstract

One factor that may influence how executive functions develop is exposure to more than one language in childhood. This study explored the impact of bilingualism on inhibitory control in autistic (n = 38) and non-autistic children (n = 51). Bilingualism was measured on a continuum of exposure to investigate the effects of language environment on two facets of inhibitory control. Behavioural control of motor impulses was modulated positively through increased bilingual exposure, irrespective of diagnostic status, but bilingual exposure did not significantly affect inhibition involving visual attention. The results partially support the hypothesis that bilingual exposure differentially affects components of inhibitory control and provides important evidence for families that bilingualism is not detrimental to their development.

**Supplementary Information:**

The online version contains supplementary material available at 10.1007/s10803-021-05234-y.

A question that all bilingual parents must answer is whether or not to pass on a second language to their children. This issue is exacerbated for parents of autistic children because while profiles vary, autism has been linked to language delays and issues with communication, making caregivers more reluctant to nurture a bilingual upbringing. However, an intriguing possibility, rarely investigated to date, is that bilingualism may be less challenging for autistic children than previously supposed and may even enhance their cognitive functioning and compensate for developmental difficulties. In this paper, we investigate the relationship between bilingual development and executive functions in autism. Below, we describe the reasons for believing that bilingualism may lead to an advantage in the development of executive functions and test this idea in a large new sample of autistic children growing up in bilingual environments.

Autism spectrum disorder (hereafter autism) is a lifelong neurodevelopmental condition estimated to affect more than 1% of the global population (MacKay et al., 2017). It is broadly characterised by two diagnostic criteria: different patterns of social communication and interactions with others, and restricted or repetitive behaviours or interests (American Psychiatric Association, APA, 2013). Autistic^1^ people are known to have highly heterogeneous individual profiles, which can include delays in language onset and use, concurrent intellectual disability and sensory atypicalities (Lord et al., 2020). These can have a broad range of consequences for daily functioning (Duncan et al., 2018), social relationships (Elmose, 2020), sensory experience and learning (Jones et al., 2020), and mental health alongside overall quality of life (Dijkhuis et al., 2017).

One area of focus for autism research is the development of, and subsequent difficulties with executive functioning (EF). Basic EF processes, including working memory and attention, interact to support aspects of higher order cognition such as complex decision making and abstract reasoning (Collins & Koechlin, 2012). One hypothesis proposes that poor cognitive control in autism impacts problem solving and is a contributing factor for differences in cognition and lifelong functioning outcomes between autistic and non-autistic people (Prior & Hoffman, 1990). Research shows difficulties with EF to be widespread in autism, including difficulties with spatial working memory (Williams et al., 2005), planning (Olde Dubbelink & Geurts, 2017), and set shifting (Hill, 2004). Moreover, repetitive movements and preference for routine that are common in autism have been associated with frontal lobe impairments, which are consistently associated with mental inflexibility (Lopez et al., 2005). Some argue that impaired EF is a key component of autism, proposing weaker coordination and integration of prefrontal executive processes as a primary dysfunction that affects other emotion and social circuits within the brain (Maximo et al., 2014). Inhibitory control is one specific and foundational EF process that is affected in autism, with differences apparent from as early as 2 years of age (St John et al., 2016).

Inhibitory control, a basic EF process, is defined as the ability to suppress thoughts or responses that are contextually learned (Anderson & Weaver, 2009). Inhibitory control can be categorised into (1) attentional interference control, and (2) prepotent response inhibition, and each type can be measured by distinct tasks (Nigg, 2000). The first refers to the capacity to selectively focus attention and suppress distracting information (Colás et al., 2017). Typically, this is measured by means of conflict resolution tasks (e.g., the Simon or Flanker Task) where response to a target objective competes with irrelevant stimuli. Results demonstrate interference effects (O’Leary & Barber, 1993) with slower response times (RTs) induced by incongruent cues relative to congruent cues, denoting a competition for attentional resources. Autistic people may have difficulty resisting interference when selectively focusing visual attention (Chevallier et al., 2013), manifested as longer reaction times and larger error rates than control groups on incongruent trials. These effects are exhibited more clearly in young children (Christ et al., 2011), suggesting a delay in development.

The second aspect of inhibitory control, prepotent response inhibition, is the ability to withhold irrelevant motor actions (Casey et al., 2001) and is classically measured through stop signal tasks (Logan, 1994). These tasks demand suppression of a learned response to a subset of stimuli and this suppression is measured by participants’ accuracy or reaction times. Although several studies report no differences between autistic and non-autistic participants groups (Ozonoff et al., 1994; Sinzig et al., 2008) on this component, others report that management of prepotent response inhibition may be impaired in autism (Johnson et al., 2007; Langen et al., 2012). A recent meta-analysis suggests that autistic people have more difficulty with both interference control (effect size = 0.31) and prepotent response inhibition (effect size = 0.55) than non-autistic people (Geurts et al., 2014), though the review also noted weaknesses in the underlying literature in terms of large variability between task procedures and dependent measures.

## Effects of Bilingualism on Inhibitory Control

A number of studies suggest that for non-autistic children, bilingualism confers advantages in the EF domain, and similar advantages are also reported for inhibitory control. Although the results are controversial (De Bruin et al., 2015), findings suggest the most robust evidence of benefits is for tasks involving the management of conflicting attentional demands, such as the Simon Task (Bialystok et al., 2005; Salvatierra & Rosselli, 2011), while no advantage is present for motor response inhibition classically associated with impulsivity (Carlson & Meltzoff, 2008; Martin-Rhee & Bialystok, 2008). The hypothesised mechanism of this effect is that bilinguals are constantly managing opposing linguistic demands, requiring application of inhibitory control (Bialystok, 2001). However, many of the research procedures lending support for the idea have been criticised (Zhou & Krott, 2016) for lack of rigour and replicability, and a number of investigations have failed to find any effect of bilingualism on inhibitory control (e.g., Arizmendi et al., 2018; Dick et al, 2019; Paap & Greenberg, 2013).

One reason for inconsistent findings is that past studies typically subdivide participants into distinct monolingual and bilingual categories for group comparisons. However, this simplistic approach disregards the variability and complexity of the bilingual experience with a range of language exposure and idiosyncratic factors impacting on neural plasticity (DeLuca et al., 2019). As there are a number of factors that produce variation of bilingual experience (e.g., number of languages spoken, age of acquisition, linguistic proficiency, switching rate in everyday life etc.), the influence of bilingualism may vary between individuals and cognitive processes (Luk & Bialystok, 2013). Another common criticism of this literature is the lack of appropriate controls (Mishra, 2018) on dimensions such as socioeconomic status and multilingualism, raising the possibility that EF skills make it easier to be bilingual, rather than the other way around.

The idea that bilingualism could enhance inhibitory control has roots in psychological theory. The adaptive control hypothesis (Green & Abutalebi, 2013) argues that language control processes adjust to meet the recurrent demands placed upon them and this is subject to context. Due to repeated language switching, dual language contexts place the highest demand on cognitive management domains within EF. This includes interference control, sustained attention and flexible switching. To compensate, these domains are likely to be enriched in terms of neural efficiency and heightened cooperation with other internal processes. Therefore, the interactional context upon which bilingualism is assessed is worth consideration and assessing participants from a range of language contexts may help establish the relationship between language learning and EF advantages. From this model, we focus on variables of cognitive control involving inhibition, and use working memory as a control variable outside this model, not anticipated to be influenced by bilingual exposure. Findings supporting enhanced working memory as a consequence of bilingualism are mixed, and some research has suggested bilingual working memory effects may not exist (Engel de Abreu, 2011) or if found are typically small in size (Grundy & Timmer, 2017). As this paper focuses on cognitive control in the form of inhibition in line with the adaptive control hypothesis, we have selected an everyday measure of working memory as a control variable only. Interestingly, recent research (Peristeri et al., 2020) has potentially implicated working memory as being enhanced through bilingualism in autistic children. However due to the nature of the task used in the study it is not clear if differences in performance can be attributed to variation in working memory capacity directly, or attributed to one of a number of separate cognitive operations working in tandem (e.g., inhibitory control, selective attention, information updating etc.).

The claim for a bilingual advantage for EF remains contentious and requires further exploration, particularly when it comes to minority populations. This study aims to contribute to the existing literature by investigating the influence of bilingual language exposure on inhibitory control in autism. Given the known inhibitory control challenges in autistic people, this investigation provides a novel insight into the relation between EF development and bilingual exposure as well as providing relevant information to practitioners and the community.

## Autism and Bilingualism

There remains widespread belief that bilingualism could overload the language development of autistic children (Hampton et al., 2017). One factor compounding this idea is specialist advice given to parents suggesting it may be safer for their child’s development to limit language to one dominant, societal language only (Kay-Raining Bird et al., 2012). Yet, the evidence that is available suggests autistic bilinguals are no more likely to experience delays or disruptions to language development over that of autistic monolingual children (Hambly & Fombonne, 2012; Lund et al., 2017). While it is true that bilingual toddlers often present with smaller vocabularies relative to monolinguals of the same age, word count does not differ in terms of total vocabulary across languages (Hoff et al., 2012), In fact, the introduction of a second language within infancy could possibly encourage imaginative play and gesture signalling (Valicenti-McDermott et al., 2013). Ultimately, there is no significant body of research evidence that contests that bilingualism is harmful for development of autistic children.

As previously discussed, research indicates difficulties with inhibitory control for autistic people, while bilingualism may confer benefits to some aspects of inhibitory control. This raises two interesting questions: could being bilingual mitigate inhibitory control difficulties for autistic children, and do varying levels of bilingual exposure directly influence inhibitory control? To date, few studies have directly considered the role of bilingualism on EF for autistic people. Gonzalez-Barrero and Nadig (2019) explored whether exposure to multiple languages could mitigate set-shifting difficulties in autistic and non-autistic children (n = 20 per group). On a computerised dimensional change card sorting task (DCCST) bilingual autistic children performed better than their monolingual autistic peers, but no effect was found from parent-report data relating to how parents perceived their autistic children to perform on everyday EF skills. Moreover, correlations between set shifting from the experimental task and set shifting observed by parents in everyday life were not significant, possibly suggesting that the DCCST fails to capture the EF demands that children face in real life. Notably, it is unclear whether these findings can be generalised to other aspects of EF. More recently, Sharaan et al. (2020) investigated the impact of bilingualism on sustained attention, working memory, interference control and task switching in Arabic-English speaking autistic and non-autistic children. When compared to monolingual participants, autistic bilingual children exhibited an advantage in sustained attention, but no difference in other measured facets of executive function.

This study has been produced as a subset of analysis from a larger longitudinal project which aims to investigate over time the effects of learning a second language on cognitive development. It is the first to focus on inhibitory control and examine bilingual exposure as a continuum in an autistic sample. Instead of categorising participants into monolingual and bilingual groups, we recruited children from a range of bilingual backgrounds to understand how different levels of bilingual exposure moderate cognitive development. We also include working memory as a control comparison not expected to be modulated by bilingualism since past research has suggested that bilingual working memory effects may not exist (Engel de Abreu, 2011) or if found are typically small in size (Grundy & Timmer, 2017). We addressed the following research questions and hypotheses:

1. How do autistic and non-autistic children perform on tasks of attentional interference control and prepotent response inhibition? Based on existing literature, it is expected that the non-autistic group will outperform the autistic group on measures of both attentional interference control and prepotent response inhibition.
2. What is the relationship between bilingual exposure and executive inhibitory processes (controlling for IQ and age) for autistic and non-autistic children? Increased bilingual exposure may be related to improved executive attentional processes for both autistic and non-autistic children. We will explore whether bilingual exposure has a similar effect for both autistic and non-autistic groups, or whether exposure closes the performance gap between autistic and non-autistic children.
3. Does bilingual exposure modulate parental reports of inhibitory control, and does this differ between participant groups? We predict a positive effect of bilingual exposure in parent reports: if bilingual exposure is positively modulating performance across EFs, we would also expect this to positively affect parental perception of their child’s EF skills. However, we do not have a strong expectation about whether this exposure effect will be reported equally in both autistic and non-autistic groups.
4. In addition, we will examine the relation between experimental and parent-report measures of inhibitory control in order to gain insights on the robustness of any findings.

## Method

### Participants

89 children (n = 38 autistic, n = 51 non autistic) participated in this study. The inclusion criteria required that all participants were exposed to more than one language, with exposure referring to individuals who spoke and/or received secondary language input at home and/or school (see Table 1 below for the participant dominant language percentage, and the Appendix for further details). However, this exposure varied widely and verbal fluency in one or more languages was not mandatory as a prerequisite for participation. Limits were not placed on IQ scores in the inclusion criteria in order to ensure a more representative autistic sample. However, IQ was included as a covariate in subsequent analyses.

**Table 1:** Descriptive overview of participants broken down by means, standard deviations and ranges

	Autistic (N = 38)			Non-autistic (N = 51)			Comparisons
	Mean	SD	Range	Mean	SD	Range	
Age (Months)	112.37	30.15	71–162	96.86	23.28	70–152	W = 689, ***p* = *0.02***
Gender	Female = 16, Male = 22			Female = 30, Male = 21			Odds ratio = 1.04, p = 1
BPVS-3	98.61	41.09	0–166	109.92	22.83	62–155	t(53.87) = 1.53, *p* = *0.13*
IQ (WASI-II Sum Raw Scores)	30.37	16.62	0–69	36.55	10.46	16–60	t(58.31) = 2.01, ***p* = *0.04***
SCQ	22.30	5.34	16–27	2.73	2.15	0–5	W = 0, ***p* = < *.001***
Dominant Language English / BILEC (%)	54.71	27.81	8–96	59.10	23.23	4–98	W = 1072, *p* = *0.39*

Bold comparisons indicate significant differences between groups at the 0.05 threshold

Comparisons are calculated using independent sample t tests for BPVS-3 and IQ. The Wilcoxon rank sum test is computed for age and the BILEC as a non-parametric alternative. Fishers exact test is computed for gender

Participating families were recruited throughout Scotland and England through a variety of methods including school networks, charity participant databases, and magazine and poster advertisements. Autistic participants were required to have a clinical autism diagnosis, confirmed by parents. As a further confirmatory measure, autistic children were also assessed on the ADOS-2 (Lord et al., 2012) and all parents completed a Social Communication Questionnaire (Rutter et al., 2003). A total of 33 children completed an ADOS-2, with five children unable to participate as they had very recently completed an ADOS-2, or due to practical constraints at home visitation. Out of this group, three children did not receive an ADOS-2 algorithm score above the likelihood threshold for a diagnosis of autism, but all of the autistic group scored above the SCQ screening threshold. All non-autistic children scored below a 7 on the SCQ, indicating that the two participant groups could be distinguished reliably by diagnostic status.

Detailed demographic information divided by Participant Group is provided in Table 1.

### Measures and Materials

Attentional interference control was measured using reaction time in the *Eriksen Flanker Task (*Eriksen & Eriksen, 1974), a computer-based spatial attention paradigm. Trials were differentiated between congruent and incongruent, with the latter corresponding to the central target facing in a conflicting direction to peripheral distractors. Reaction times of correct responses were recorded, as output of accuracy has been shown to lead to ceiling effects, exhibiting a reduced variance in collected data (e.g., Duthoo et al., 2014). Cartoon fish stimuli (height: 2 cm) acted as both the target and distractor stimuli for this task, identical apart from pointed gaze direction and screen position. Longer reaction times indicate poorer attentional interference control.

Prepotent response inhibition was measured by accuracy (specifically, false start rate) on the *Psychomotor Vigilance Task* (Dinges & Powell, 1985) through inaccurate button presses indicating the presence of a target. A red stopwatch (height: 1.8 cm) positioned on the centre of a screen was presented as the target stimulus. The timer counts with numerals upwards in milliseconds before disappearing and being replaced by a reaction time speed, upon the participant responding to the stimulus accordingly. Lower false-positive rates indicate better prepotent response inhibition. A 15-inch Laptop was used to administer both the psychomotor vigilance and flanker task. Inquisit 5 experiment generator software (2016) presented the stimuli and recorded participants’ responses.

EF in the home environment was measured using a parental questionnaire; the *Behavior Rating Inventory of Executive Function Second Edition* (Gioia et al., 2000). The questionnaire comprises 63 items scored as: often, sometimes and never. The output can be divided into Behavioural Regulation Scales (Inhibit, Shift and Emotional Control) and Metacognition scales (Initiate, Working Memory, Plan/Organise, Organisation of Materials, and Monitor). Higher scores indicate increased levels of executive dysfunction in their respective domain. Only Inhibit and Working Memory subscale scores were analysed in this study.

The bilingual experience of participating children was captured by the short version of the *Bilingual Language Exposure Calculator* (*BILEC*) parent questionnaire (Unsworth, 2013). Language exposure was measured by the number of hours each language was used at home, (including after school, at weekends, and during the holidays) during the school day, and with friends. By placing participants on a continuum of language exposure, it was possible to directly compare experience without the loss of variance caused by splitting participants into distinct exposure level groups. Bilingual exposure was calculated from the Bilingual Language Experience Calculator as a percentage ranging from 0 to 100% and measured only through language input.

In order to gather a measure of IQ, participants completed the *Wechsler Abbreviated Scale of Intelligence II* (WASI-II; Wechsler, 2011). Only the vocabulary (31 items) and matrix reasoning (30 items) subtests were used, sufficient to calculate a partial IQ score and as an estimate of general cognitive ability. IQ limits were not stated within the inclusion criteria in order to permit a representative autistic sample. However, IQ was included as a covariate in subsequent analyses along with age.

Receptive vocabulary was assessed using the British Picture Vocabulary Scale Third Edition (Dunn & Dunn, 2009). The assessment measures age-appropriate receptive vocabulary abilities. Participants are instructed to match a word spoken by the examiner to one of four pictures using non-verbal responses.

The Autism Diagnostic Observation Schedule, 2nd edition (ADOS-2; Lord et al., 2012) is a standardised assessment tool that is semi-structured. It is used to measure social and communication behaviours which contribute towards a diagnosis of autism. Activities are administered from one of four modules. Modules are selected as a result of language and developmental level.

The SCQ-Lifetime (Rutter et al., 2003) is a parent-administered questionnaire which may function as an initial screening measure for autism. This measure accounts for the developmental history of the individual. Scores greater than 15 suggest higher-than-average levels of autistic traits.

### Procedure

Ethical approval was obtained by the University of Edinburgh Psychology Research Ethics Committee (336-1718/5). Informed consent from parents and caregivers was recorded electronically before the appointment and signed physically on the day of assessment. Children were also asked to provide verbal assent prior to participation. Data was collected in a single session by the research team. Data was collected through two-hour home visits so as to maximise ecological validity and ensure time for a break. As a reward, children received a bag of sensory toys upon completion of the session.

Within the task battery, most participants first completed the Psychomotor Vigilance Task and then immediately after, the Flanker Task. Parents completed the BRIEF within two weeks prior to the home visit. Full detail on the administration protocol for the Flanker and Psychomotor Vigilance tasks are included in the Supplementary Appendix Materials.

### Scoring and Analysis

Flanker Task data were processed prior to analysis to exclude trials in which participants scored inaccurately (13.84%) or responded within 200 ms or less (0.12%). PVT data were not treated prior to analysis. For all independent variables, data were standardized to have a mean of 0 and a standard deviation of 1, allowing all analyses to be compared on the same scale.

To capture individual variability, we used mixed effects modelling to examine the influence of bilingual exposure on inhibitory control tasks. We used backwards selection to find the best fitting regression models for our data, beginning with models that incorporated all variables as fixed effects. For mixed models, we also incorporated by-participant random intercepts. We removed predictor variables through a backwards stepwise selection procedure based on the model that favoured the Akaike Information Criterion (AIC). Marginal R squared for fixed effects, and where appropriate conditional R squared for both fixed and random effects, were calculated for each final model to act as a measure of model fit and variance explained. Our only constraint on model selection was the conservative decision to always incorporate Age and IQ into the regression, as these variables were not matched between participant groups, and each can potentially modulate task performance. All analyses were conducted in R with mixed models fit using the lme4 package (Version 3.3.1; Bates et al., 2015).

For each experimental task, our initial mixed effects model included fixed effects of Group (autistic/non-autistic), Bilingual Exposure, Age and IQ. For the Psychomotor vigilance task, where the outcome variable (false starts) was binary, we used a mixed logistic regression, and incorporated a two-way interaction term between Participant Group and Bilingual Exposure. For the Flanker Task, where RTs was the outcome variable, we used a mixed linear model, and included an additional predictor of Trial Type (congruent/incongruent) with a three-way interaction term between Participant Group, Bilingual Exposure and Trial Type. Both models additionally included by-participant random intercepts.

Due to the standardised scoring of the BRIEF, simple multiple linear regression was deployed. BRIEF Inhibit and Working Memory subscale scores were fitted with multiple linear regression using Participant Group (autistic/non-autistic), Bilingual Exposure, Age and IQ as independent variables. Also included was a two-way interaction term between Participant Group and Bilingual Exposure.

We used first order Spearman correlations (robust to the presence of extreme values) to test associations between parent reports and EF from the experimental tasks. As multiple analyses were run, a Bonferroni adjustment was applied (alpha value of 0.05/8 = 0.006).

Underlying assumptions were checked and validated for all analyses. The Flanker Task violated the assumptions of normality and homogeneity of variance. To resolve, outliers were removed, using a cut-off threshold of 2.5 standard deviations from the mean. Different transformations of the dependent variable were then tested with a log transformation selected as the most appropriate adjustment. Upon being rechecked, data passed visual checks for normally distributed residuals and demonstrated constant variance. The pattern of results reported here was the same when run with unadjusted Flanker Task data.

For the PVT Task outliers were similarly removed, considered as observations greater than or equal to Cook’s D of 4/n and calculated from simple logistic regression. Upon visual inspection only influential values were removed by this process.

For BRIEF analyses outliers were removed, specified as greater than or equal to Cooks D of 4/n with additional visual leverage checks undertaken. Results further excluded 3 participants whose parents failed to score as Acceptable on the BRIEF Inconsistency Scale.

## Results

A summary of descriptive statistics for the participant sample is provided in Table 2.

**Table 2:** Descriptive statistics with means and standard deviations for all measures of executive functioning

Experimental tasks	Non-Autistic		Autistic	
	Mean	SD	Mean	SD
FT	N = 46		N = 28	
Accuracy error	10.10	14.31	21.66	25.51
Reaction time	995.64	482.65	879.76	426.32
PVT	N = 37		N = 29	
Total false starts	8.35	10.76	7.81	13.27
BRIEF				
Inhibit	N = 47		N = 27	
Subscale scores	11.17	2.78	17.59	4.01
WM	N = 47		N = 25	
Subscale scores	10.61	2.49	16.92	3.86

### Flanker Task

The best fitting model incorporated predictors for Age, IQ, Bilingual Exposure and Trial Type with a summary presented in Table 3. Increased Age and IQ were associated with shorter RTs, whereas greater bilingual exposure resulted in marginally longer RTs. Incongruent trials elicited longer RTs than congruent trials. Participant Group was not included in the best model, nor did it have a significant effect.

**Table 3:** Fixed and random effect structure as a summary of the Flanker Task linear mixed model

Predictors	Log transformed reaction times			
	Estimates	CI	p	DF
(Intercept)	6.72	6.66 to 6.78	** < 0.001**	73.03
Trial Type [incongruent]	0.12	0.10 to 0.14	** < 0.001**	2928.48
Age	− 0.08	− 0.15 to − 0.00	**0.040**	69.90
IQ	− 0.16	− 0.24 to − 0.09	** < 0.001**	70.97
Bilingual exposure	0.06	− 0.00 to 0.12	0.051	70.20
Random effects				
σ^2^	0.08			
τ_00_	0.06			
ICC	0.46			
N	74			
Observations	2988			
Marginal R^2^/Conditional R^2^	0.27/0.60			

Bold comparisons indicate significant differences between groups at the 0.05 threshold

The conditional R^2^ of 0.60 accounts for the variance explained by the whole model, where the marginal R^2^ of 0.27 reflects only the contribution of the fixed effects

No higher-order interactions were identified as significantly predicting reaction time, meaning that the difference between congruent and incongruent trials did not significantly vary based on Age, IQ, or Bilingual Exposure. Bilingual exposure was significant using approximate p-values (0.047), however upon calculating more precise p values based on conditional F-tests with Kenward-Roger approximation for the degrees of freedom, bilingual exposure failed to reach significance (0.051).

### Psychomotor Vigilance Task

**Fig. 1:**
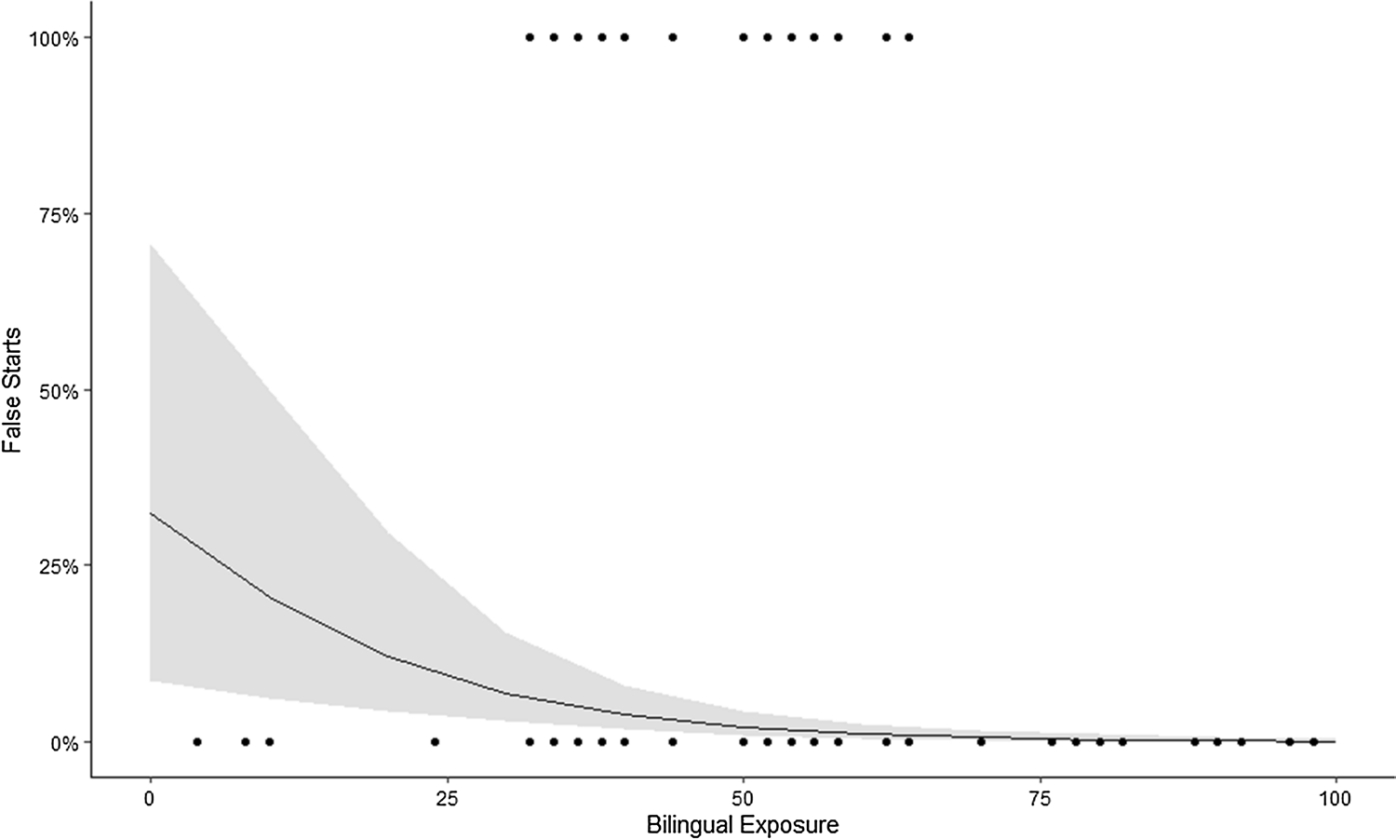
Marginal effects of the mixed effects logistic regression model. This shows the probability of a participant producing a false start on the Psychomotor Vigilance Task in conjunction with Bilingual Exposure level

The best fitting model incorporated Bilingual Exposure, Age, and IQ as significant predictors on the likelihood of producing a False Start. A summary of this model is shown in Table 4. Increased Bilingual Exposure (see Fig. 1) and Age were associated with a lower likelihood of false start rate. Higher IQ was associated with a higher likelihood of false start button presses. Again, Participant Group was not found to be significant, nor included in the best model.

**Table 4:** Fixed and random effect structure as a summary of the Psychomotor Vigilance Task mixed effects logistic regression

Predictors	False starts		
	Odds ratios	CI	p
(Intercept)	0.01	0.01–0.03	** < 0.001**
Age	0.41	0.20–0.83	**0.014**
IQ	2.46	1.22–4.99	**0.012**
Bilingual exposure	0.19	0.11–0.34	** < 0.001**
Random effects			
σ^2^	3.29		
τ_00_	3.75		
ICC	0.53		
N	66		
Observations	2828		
Marginal R^2^/Conditional R^2^	0.28/0.66		

Bold comparisons indicate significant differences between groups at the 0.05 threshold

*An odds ratio greater than 1 describes a positive relationship between variables whereas an odds ratio less than 1 describes a negative relationship

### Parent-Reported EF

**Fig. 2:**
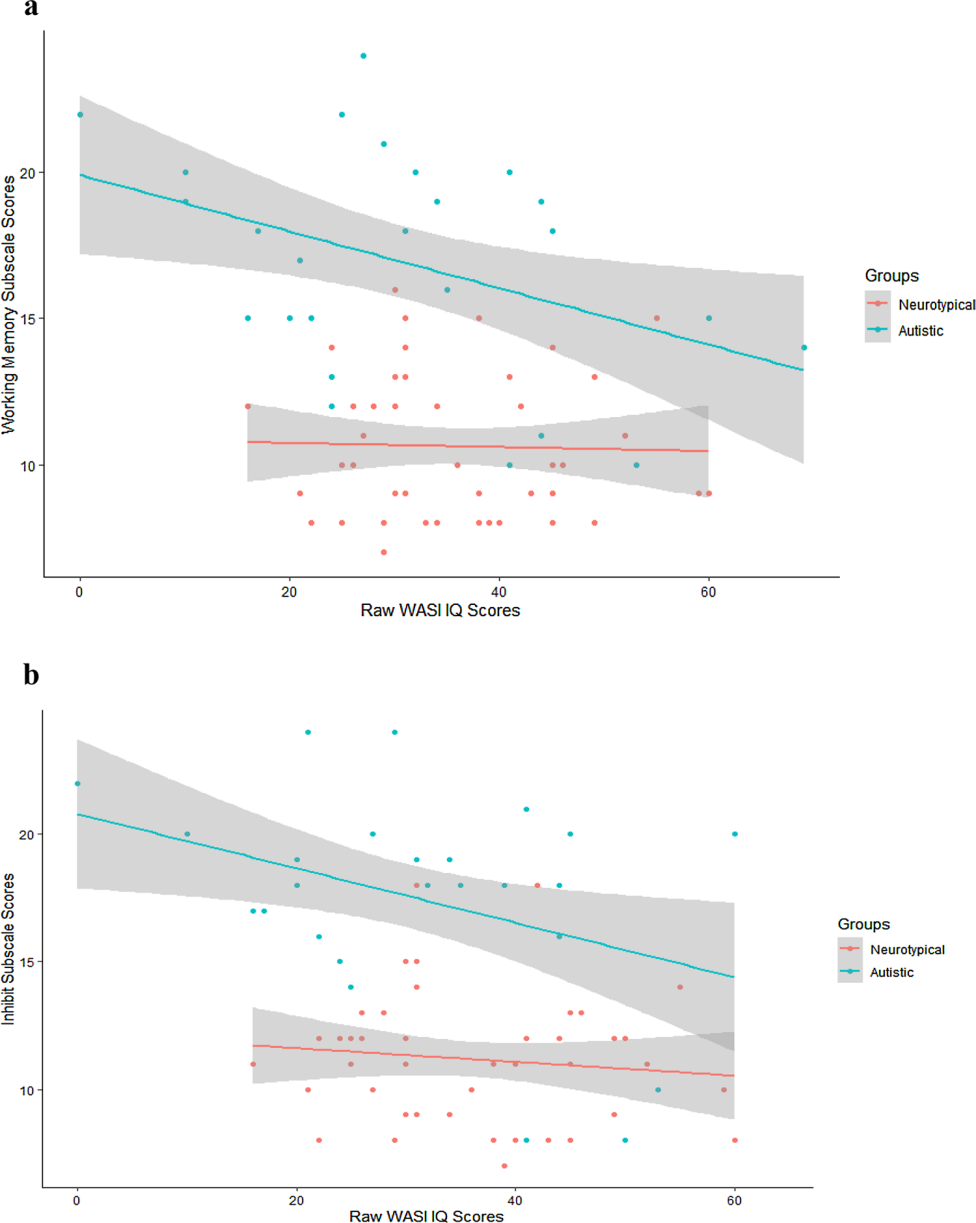
**a** Main effect of multiple regression analysis on working memory scores from the BRIEF questionnaire, where IQ scores were the only significant predictor. **b** Shows the main effect of multiple regression on the Inhibit subscale of the BRIEF, where IQ scores were the only significant predictor

A multiple regression (n = 74) was run to predict inhibitory control scores using Age, IQ, Participant Group, and Bilingual Exposure. Only IQ and Participant Group were selected as predictors in the best model which significantly predicted inhibitory control scores, *F*(*2,71*) = *36.90*, *p* = *0.00*, *R*^*2*^ = *0.51*. An identical control model (N = 74) was run with Working Memory scores as the dependent variable. The best model predicted Working Memory scores*, F*(*2,71*) = *41.58*, *p* = *0.00 R*^*2*^ = *0.54,* with IQ and Participant Group significantly contributing as independent variables. In each model, lower IQ corresponded to higher BRIEF scores (more executive dysfunction), and non-autistic participants’ scores suggested higher levels of executive function skills than the autistic group. Results from both models are presented in Table 5 and illustrated in Fig. 2a, b.

**Table 5:** Summary of two BRIEF multiple regressions using inhibit and working memory subscale scores as dependent variables

Predictors	Model summary			
	EST	SE	T	p
Inhibit scores				
Intercept	11.30	0.47	24.02	**0.00**
WASI-II IQ	− 0.91	0.42	− 2.17	**0.03**
Participant Group (ASD)	6.00	0.80	7.54	**0.00**
Working memory scores				
Intercept	10.75	0.45	23.84	**0.00**
WASI-II IQ	− 0.94	0.40	− 2.32	**0.02**
Participant Group (ASD)	6.10	0.76	7.99	**0.00**

Bold comparisons indicate significant differences between groups at the 0.05 threshold

### Correlations

Spearman correlations assessed the strength of relationship between EF in experimental tasks alongside BRIEF scores. Table 6 presents the results of the correlations. BRIEF questionnaire Inhibit and Working Memory subscale scores are correlated with accurate incongruent Flanker Task reaction times and the total number of false starts, listed by group. No correlations are significant upon applying a Bonferroni correction.

**Table 6:** Spearman correlations between measures of executive functioning

	Correlations	
	Flanker task RTs	PVT false starts
Non-autistic		
Inhibit scores	0.14	0.19
WM scores	0.00	− 0.15
Autistic		
Inhibit scores	0.49	0.84
WM scores	0.33	− 0.10

## Discussion

This is the first study to explore the continuous effect of bilingual exposure on attentional interference control and prepotent response inhibition in autistic and non-autistic children. We examined the relationship between bilingual exposure and executive inhibitory processes captured by experimental tasks and via parent report and were particularly interested in investigating the effects of bilingual exposure in an autistic sample. We discuss four key findings: (1) Successful prepotent response inhibition is reliably predicted by language exposure level whereas attentional interference control is not; (2) A group effect on EF task performance was not found; (3) Parental reports of children’s inhibitory control in everyday life was not modulated by bilingual exposure; (4) Participant performance on experimental tasks was not correlated with parent-report measures capturing everyday functioning.

### Experimental EF Tasks

A main aim of this investigation was to explore the relationship between bilingualism and executive inhibitory processes in autistic and non-autistic children. The results suggest that accurately managing behavioural inhibition of motor functions is modulated through bilingual language use: the probability of initiating a PVT false start on an experimental trial was reduced by increasing bilingual exposure and this effect was consistent regardless of autism diagnostic status. We also found a strong correlation between parent reports of inhibitory control and PVT false start rate, but this finding was not significant. However, analysis suggests that bilingual exposure does not meaningfully impact inhibitory thought processes when it comes to visual selection and the suppression of irrelevant information. Although an effect of increasing bilingual exposure was included in the best FT model as slowing reaction time of attentional control, the factor is non-significant. In addition, the absence of group effects on performance on experimental tasks was surprising and may suggest that the lack of group difference is a result of bilingualism mitigating EF impairments for autistic children. Our results were similar to that of Sharaan et al. (2020) who also found no effect of diagnosis group on PVT false start rate. However, more surprising was that their study found no main effect of language group on false start rate, although they did identify an interaction where autistic bilinguals showed significantly lower mean false starts than autistic monolinguals. This is somewhat in contradiction to the results of our study where bilingual exposure increased performance regardless of diagnostic status. The reason for the discrepancy could be due to a number of factors including power and approach to analyses (mixed modelling vs ANOVA), differences in group conceptualisation (bilingual exposure vs monolingual/bilingual groups) or IQ inclusion criteria (no exclusion vs only participants who score as intellectually average or above).

Why might bilingualism interact with one aspect of inhibitory control but not another? Generally, it is agreed that inhibitory control is not a cohesive construct, but instead a collection of cognitive processes grouped by a shared function (Roberts et al., 2011). Differences between these cognitive processes exist in terms of how mental processes are applied to tasks, and in brain circuitry (Mostofsky et al., 2003), thus explaining why bilingualism may modulate operations separately. The two functions can be teased apart in a number of ways. First, inhibitory control involving oculomotor response and deployment of visual attention (as in the Flanker Task) is executed independently of inhibitory control involving more physical motor regulation (as in the PVT) (Aron et al., 2004). Second, selective filtering of attention whilst overlooking irrelevant distractors (as in the Flanker Task) is goal directed and therefore requires top-down processing (Pinto et al., 2013), whereas regulation of impulses (as in the PVT) is typically automatic, incorporating some elements from bottom-up processes. Lastly, conflict tasks like the Flanker Task have a unique relationship with working memory (Carlson et al., 2002) that is not present with delay tasks like the PVT, in the sense that perceptual features are held and internally manipulated via the selection of a desired mental representation. Consequently, it is possible that our two measures are underpinned by distinct cognitive mechanisms that interact with bilingualism differently.

Our findings suggest that any bilingual advantage for inhibitory control is evident under specific conditions only: namely through the emergence of sustained attention where the behavioural regulation of motor impulses is required. In contrast, our results cast doubt on the idea that bilinguals’ exercise of inhibitory control, via activation of a target language and suppression of a non-context appropriate language, benefits selective visual attention. The findings accentuate the need to use multiple measures when evaluating a cognitive ability as a means of gathering converging evidence.

These results do not contradict the possibility that children with stronger executive skills may be more likely to learn multiple languages, and thus offer more positive response overall to bilingual input, explaining potential biases in our sample. However, our inclusion criteria were as inclusive as possible, including children with learning disabilities and children with a wide range of language competency, giving some credence to a causal relationship between bilingual exposure on EF. A more counterintuitive finding is that as the IQ of participants increased, the likelihood of producing false starts also increased. Researcher observations during data collection suggest that children with higher IQs may have been more engaged with the PVT task in general, and as a result prone to accidental button presses through trying to predict when the target would appear as quickly as possible. In contrast, children with lower IQs demonstrated less engagement during data collection and would often look away from the task until the target was clearly fixed on the screen, despite previously being told they should complete the task as quickly and accurately as possible. This result would perhaps not have arisen had we implemented an inclusion criterion based on IQ above a certain threshold. Furthermore, although the WASI-II has been found to characterise meaningful construct validity (Canivez et al., 2009), caution must be taken so as not to over-interpret the results from a brief and albeit rapid estimate of general functioning which cannot replace a full intellectual quotient.

The hypothesis that non-autistic people outperform autistic peers on measures of attentional interference control and prepotent response inhibition is not supported by the results. Here, we find no significant difference in performance between the two groups on either the FT or PVT. This is despite substantial evidence and consensus within the academic community that executive dysfunction is widespread in autism and may underlie the core presentation used in diagnosis (e.g., Russell, 1997; Xie et al., 2020). For example, various studies indicate that autistic children perform poorly on distractor interference (e.g., Geurts et al., 2008) and on prepotent response inhibition (e.g., Bíro & Russell, 2001), with the latter being linked to repetitive and stereotyped behaviour (Schmitt et al., 2017).

The fact that an autistic disadvantage is often found in tasks of attentional interference control and prepotent response inhibition, but not in our sample of participants subject to bilingual exposure, may suggest a bilingualism effect compensating autistic difficulties in EF. This supports the idea that bilinguals’ regular engagement with inhibitory thought processes and language switching can be protective against behavioural and cognitive inflexibility (Faja & Darling, 2018). Yet, this finding is not replicated in terms of parent perception of EF—in this case significant relations were found with autism diagnostic status and IQ only. Still, various studies have suggested that caregivers may not always measure the skills of their children accurately (Mattson et al., 2020) and uniting the differences between experimental outcomes and parental perception continues to pose problems for forging consistent patterns of results.

### Further Considerations and Limitations

The results of this study are specific to considering bilingualism as exposure to more than one language in terms of listening and should be generalised as such. The decision was taken to include only bilingual input into analyses, rather than output, because the autistic participants in this study encompass a considerable range in ability level, from highly verbal to minimally verbal with learning difficulties. Certainly, it is possible that listening to a second language demands fewer mental resources than actively speaking and this could be expected to confer different or weaker cognitive benefits. In this context, the observed impact of bilingual exposure on prepotent response inhibition in our data is perhaps surprising, and important.

One potential drawback of our decision to study bilingualism as a continuum, rather than through the traditional design of comparing bilingual and monolingual groups, is that a latent effect of bilingualism could be biasing the results in the sense that multiple language exposure may have enhanced EF throughout the sample. A further limitation is that our sample didn’t include trilinguals. Remarkably, trilinguals have demonstrated several distinct cognitive differences compared to bilinguals. For example, trilingual adults are less likely to develop a mild cognitive impairment compared to bilinguals and therefore may be bettered safeguarded against cognitive aging (Perquin et al, 2013). Could then a trilingual group have produced different results to our recruited bilinguals? Possibly, but it has already been shown that while outperforming monolinguals, trilinguals do not show an advantage on the Flanker Task compared to bilinguals (Schroeder & Marian, 2017). Nevertheless this would have been interesting to capture with the inclusion of the bilingual exposure effect found in the PVT. Lastly, standardised clinical assessments may have provided more valid and replicable results. However, the measures for this project were adapted from a larger study which aimed to engage non-autistic and autistic participants alike through activities in their own home, and therefore the measures used was not purely clinical.

Future research related to inhibitory control should clarify the mechanism underlying the engagement and disengagement of attention. Otherwise, investigation is still required to ascertain the effects of bilingual exposure on various aspects of EF for autistic children as at this time such literature is scarce and our findings cannot be generalised globally.

## Conclusions

We find no evidence that autistic children are adversely affected by bilingual exposure. In fact, bilingualism may benefit certain aspects of inhibitory control, namely motor impulsivity, and this applies for both autistic and non-autistic children. This research contributes to the evidence base for families with autistic children. Caregivers should be informed about the effects on development of incorporating more than one language into a child’s linguistic environment. There is also a need to inform professionals working with autistic children and their families, whose advice is influential on parental decision making. Finally, since executive functioning is a significant aspect of cognitive development, this study considering the intersecting influences of bilingualism and autism contributes to our interpretation of how executive functioning is shaped by clinical and environmental factors.

## Supplementary Information

Below is the link to the electronic supplementary material.

Supplementary file1 (DOCX 53 kb)

## Footnotes

1 There is debate regarding the language used when describing autism. Here we use identity-first language (e.g., “autistic person”) as opposed to person-first language (e.g., “person with autism”) to respect the preference of the majority of autistic people (see Gernsbacher, 2017; Kenny et al., 2016).
