# Supplementary material for: Measuring the Impact of Bilingualism on Executive Functioning Via Inhibitory Control Abilities in Autistic Children
Source: J Autism Dev Disord. 2021 Aug 18;52(8):3560–73. doi: 10.1007/s10803-021-05234-y (PMC9296418; doi:10.1007/s10803-021-05234-y)
Supplement: Supplementary file 1 — Supplementary file1 (DOCX 53 kb) [file 10803_2021_5234_MOESM1_ESM.docx]

Appendix Materials

Bilingualism

The bilingualism variable described in this study was produced from the following: taking the smaller of the two input percentages (language listened to at home and at school) as calculated by the Bilingual Language Exposure Calculator and multiplying by two. Therefore, bilingualism was measured upon a single percentage scale upon which participant’s exposure level can be compared. The calculation acts as an elegant reduction and simplification from the four percentages produced by the Bilingual Language Exposure Calculator (two for input and two for output) into a single identifiable number. Throughout analysis, bilingualism is kept as a continuous percentage from 1 to 100 without being dichotomised into levels.

Inclusion criteria required that all participants were exposed to more than one language, with exposure referring to individuals who spoke and/or received secondary language input at home and/or school. Secondary languages were not restricted to a specific region of the world and were globally widespread within the participant sample. Also, for some of the participants the secondary language was English, and the parent’s native language was used more regularly.

Executive Function Assessments

The research team verbally explained the tasks to the participants and confirmed their understanding of the overall procedure. Participants were instructed for the Flanker Task to place separate index fingers on the keyboard letters E and I in preparation for upcoming trials. Each trial demanded a button press response dependent on the gaze direction of a central cartoon fish stimulus within a horizontal array consisting of five fish. The E button indicated a left looking central fish and the I button indicated right. Participants were asked to ignore the other four fish in the array as these acted as distractors and would each randomly face congruent or incongruent with the gaze of the middle target. A feedback screen revealing “Try again!” shown after the target image was given if participants responded incorrectly, or warning “Too slow!” if participants did not respond in time. See Figure 2 for an example.

The Flanker Task started with 20 practice trials displaying an easier version of the task where only the target fish is presented, followed by an additional 20 practice trials where participants responded to images of the target fish and four flanker distractors. After the practice trials participants underwent 120 test trials split in half by a 15 second break. Test trials were presented in random order sampled with 30 trials per condition: congruent, incongruent, left looking target and right looking target. Trials terminated after a response, or after 3000ms without a response. The interval between a response and the subsequent presentation was 1500ms, and any error feedback screens within trials lasted for 1000ms.


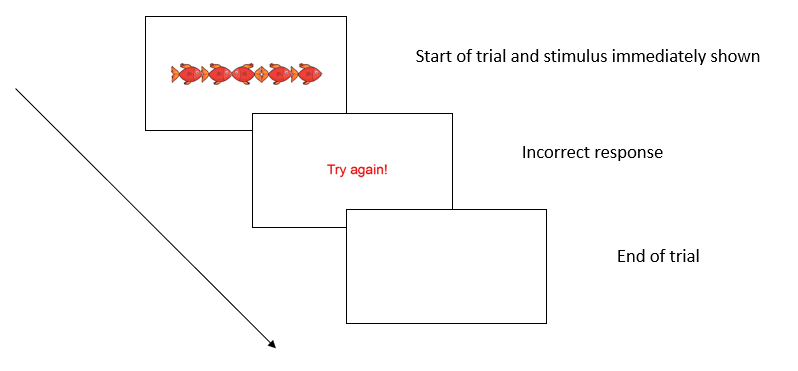


**Figure 1:** A trial sequence from the Flanker Task. This is an incongruent trial as demonstrated by the contrast in gaze between the middle fish target and distractors.

Upon the start of a Psychomotor Vigilance Task trial, a blank screen would be presented on the computer monitor. A red stopwatch would randomly appear at an interval unknown to the viewer. Participants would respond by pressing the spacebar as quickly as possible as soon as the target was sighted. Before the start of the next trial, a screen showing the reaction speed in milliseconds from onset of the target stimulus to response would display on the screen. Responses made when a target was not present were fed back to participants with a warning message “too fast!” or “too slow!”.

The experimental sequence of the Psychomotor Vigilance Task first showed a habituation phase lasting approximately 1 minute, which was not collected for analysis. The test phase then lasted for six minutes. Each trial followed a sequence in which a target appeared on a blank screen randomly between 2-10 seconds. This was followed by a reaction time feedback screen lasting 1000ms. The only deviation to this trial sequence was a false start response warning appearing on the screen lasting 300ms. An illustration of a trial is pictured in Figure 1.


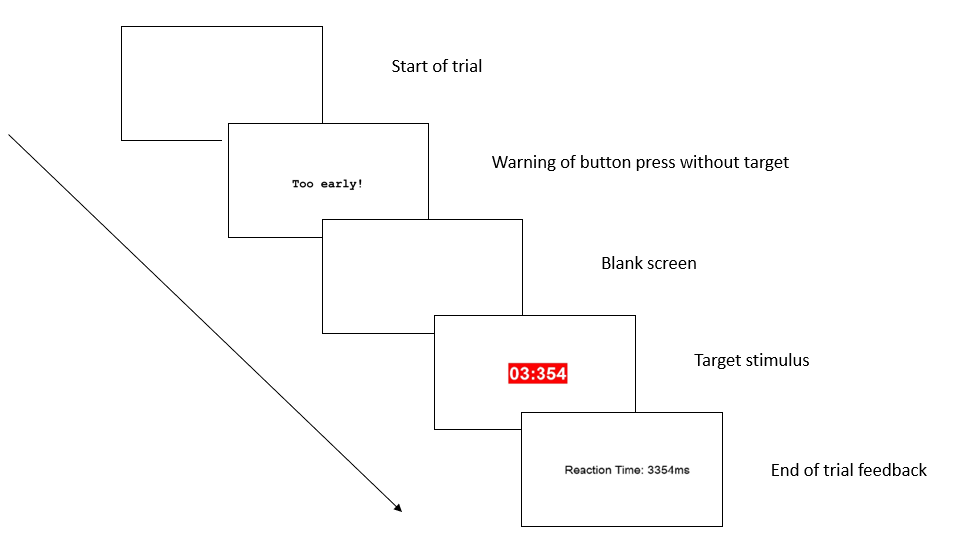


**Figure 2:** A trial sequence demonstrating the Psychomotor Task. This is an example of a participant responding too quickly (false start) and being forced to wait for the target to appear. The arrow designates the order of which the presentation is shown.
